# Simultaneous measurement of nascent transcriptome and translatome using 4-thiouridine metabolic RNA labeling and translating ribosome affinity purification

**DOI:** 10.1093/nar/gkad545

**Published:** 2023-06-28

**Authors:** Hirotatsu Imai, Daisuke Utsumi, Hidetsugu Torihara, Kenzo Takahashi, Hidehito Kuroyanagi, Akio Yamashita

**Affiliations:** Department of Investigative Medicine, Graduate School of Medicine, University of the Ryukyus, Nishihara-cho, Okinawa 903-0215, Japan; Department of Dermatology, Graduate School of Medicine, University of the Ryukyus, Nishihara-cho, Okinawa 903-0215, Japan; Department of Biochemistry, Graduate School of Medicine, University of the Ryukyus, Nishihara-cho, Okinawa 903-0215, Japan; Department of Dermatology, Graduate School of Medicine, University of the Ryukyus, Nishihara-cho, Okinawa 903-0215, Japan; Department of Biochemistry, Graduate School of Medicine, University of the Ryukyus, Nishihara-cho, Okinawa 903-0215, Japan; Department of Investigative Medicine, Graduate School of Medicine, University of the Ryukyus, Nishihara-cho, Okinawa 903-0215, Japan

## Abstract

Regulation of gene expression in response to various biological processes, including extracellular stimulation and environmental adaptation requires nascent RNA synthesis and translation. Analysis of the coordinated regulation of dynamic RNA synthesis and translation is required to determine functional protein production. However, reliable methods for the simultaneous measurement of nascent RNA synthesis and translation at the gene level are limited. Here, we developed a novel method for the simultaneous assessment of nascent RNA synthesis and translation by combining 4-thiouridine (4sU) metabolic RNA labeling and translating ribosome affinity purification (TRAP) using a monoclonal antibody against evolutionarily conserved ribosomal P-stalk proteins. The P-stalk-mediated TRAP (P-TRAP) technique recovered endogenous translating ribosomes, allowing easy translatome analysis of various eukaryotes. We validated this method in mammalian cells by demonstrating that acute unfolded protein response (UPR) in the endoplasmic reticulum (ER) induces dynamic reprogramming of nascent RNA synthesis and translation. Our nascent P-TRAP (nP-TRAP) method may serve as a simple and powerful tool for analyzing the coordinated regulation of transcription and translation of individual genes in various eukaryotes.

## Graphical Abstract

**Graphical Abstract:**
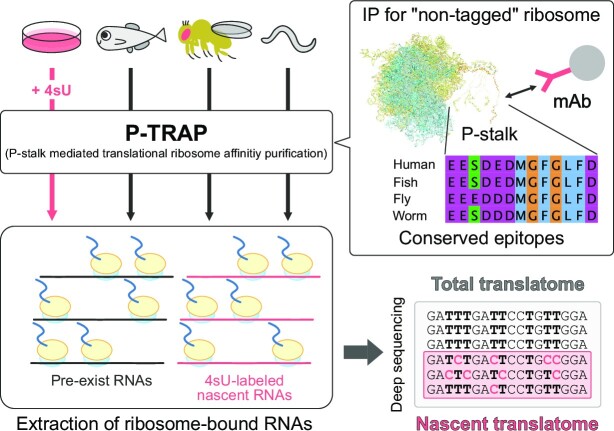

## INTRODUCTION

The regulation of gene expression plays a pivotal role in diverse biological and physiological processes such as cell differentiation, development, environmental responses, and immune responses. One determinant of gene expression is the amount of mature RNA, which reflects the balance between RNA synthesis and degradation (1–5). To understand the dynamics of RNA synthesis and degradation comprehensively, metabolic RNA labeling techniques using nucleotide analogs have been developed (6–14). For example, thiol (SH)-linked alkylation for the metabolic sequencing of RNA (SLAMseq) enables to uncover 4-thiouridine (4sU)-labeled transcripts in cDNA libraries by bioinformatic detection of specific T-to-C (T>C) conversions at the sites of 4sU incorporation (9). Combining SLAMseq with QuantSeq, a deep sequencing close to the 3′ end of polyadenylated RNAs (15), allows rapid and quantitative access to 4sU-labeled transcripts expression profiles (9). These techniques have addressed the dynamic aspects of gene expression regulation in various biological processes such as RNA quality control, oncogenesis, and embryogenesis (16–18).

The final output of gene expression, which indicates the expression level of proteins, is significantly influenced by RNA translation. Several studies have reported that the primary set of RNA transcripts obtained by standard RNA-seq experiments may have a low quantitative correlation with the proteome (4,19–21). To overcome this issue, analyzing the translatome, which refers to mature RNAs bound to ribosomes for protein synthesis, helps in estimating individual gene expression levels (22–24). In the standard polysome profiling technique, ribosome-bound RNAs are fractionated using sucrose density gradients and analyzed using RNA-seq (25,26). Translating ribosome affinity purification (TRAP) is also used to analyze translatomes which requires the expression of affinity-tagged ribosomal proteins of the large (60S) ribosomal subunit by genetic modification of cells and organisms. The cell type-specific promoter controls the expression of affinity-tagged ribosomal proteins, allowing the capture of ribosome-bound RNAs from specific cells/tissues in organisms (22,27–29). Following the immunoprecipitation of affinity-tagged ribosomes, ribosome-bound RNA was analyzed using RNA-seq. In a recently established ribosome profiling (Ribo-seq) technique, RNase digestion of ribosome-bound RNAs resulted in ribosome-protected RNA fragments (RPFs). The RPFs are converted to a library for deep sequencing to determine the precise position and density of ribosomes at nucleotide resolution (30–32).

Although the regulation of RNA synthesis, degradation, and translation are distinct steps, these control interplay in gene expression (33,34). The simultaneous measurement of these different controls takes advantage of a comprehensive understanding of complicated gene expression regulation. Recently, nascent Ribo-seq (nRibo-seq) was developed to simultaneously measure nascent RNA synthesis and translation by combining Ribo-seq with 4sU metabolic RNA labeling (35). nRibo-seq estimates whether a short RPF is derived from a 4sU-labeled nascent RNA by a binomial distribution and simultaneously measures the RNA synthesis rate and translation efficiency at the level of bulk or specific RNA groups. This pioneering technique revealed variable ribosome loading rates among the functional gene subsets. However, Ribo-seq deals with the short length of RPFs, making the reliable quantification of 4sU incorporation for individual genes difficult (35).

In this study, we developed a simple method for the simultaneous measurement of nascent RNA synthesis and translation at the gene level by combining the TRAP technique with 4sU metabolic RNA labeling. Since TRAP captures full-length RNAs bound to the ribosomes, it is compatible with QuantSeq, which analyzes the inherently uridine-rich 3′ UTRs of polyadenylated RNAs, facilitating the robust quantification of T>C conversions. In contrast to the conventional TRAP technique that requires genetically engineered cells, we developed an endogenous ribosome affinity purification method using a monoclonal antibody against an evolutionarily conserved ribosomal P-stalk protein. The P-stalk mediated translational ribosome affinity purification (P-TRAP) technique enables convenient access to the translatome in diverse eukaryotes, including mammals, fish, insects and nematodes, without genetic manipulations. We applied a combination of conventional TRAP or the newly developed P-TRAP with 4sU metabolic RNA labeling and defined the nascent RNA synthesis and translation at the level of individual genes in the acute unfolded protein response (UPR) within the endoplasmic reticulum (ER). This simple and versatile translatome analysis technique would enhance our understanding of the complex regulation of gene expression in various eukaryotes.

## MATERIALS AND METHODS

### Plasmid and antibodies

A plasmid for the stable expression of enhanced green fluorescent protein (EGFP) fused to the N-terminus of the ribosomal protein L10a (EGFP-L10a) in mammalian cells was constructed using standard procedures. Briefly, a synthetic DNA fragment of EGFP-L10a was cloned into an expression vector pcDNA5/FRT/TO (V652020, Thermo Fisher Scientific). Although the human ribosomal protein L10a is referred to as uL1 in standard nomenclature (36), we used L10a here following the development of the TRAP method.

A homemade anti-ribosomal protein P0 monoclonal antibody (clone 9D5) was kindly provided by Dr. Toshio Uchiumi and Dr. Hiroe Sato (Niigata University) and is now available from MBL (RN004M, MBL). The isotype control antibody for 9D5 (mouse IgG2a κ) was purchased from Proteintech (65208-1-IG). Antibodies against ribosomal proteins uL3 (GTX114725, GeneTex), L10a (A305-061A, BETHYL), uS2 (GTX114734, GeneTex), uS3 (GTX103964, GeneTex), uS15 (GTX101839, GeneTex) and PABP4 (A301-467A, BETHYL), CBP80 (A301-793A, BETHYL) and GAPDH (M171-7, MBL) were purchased commercially. A homemade anti-eIF4A3 polyclonal antibody was prepared as described previously (37).

### Cell lines and animals

Flp-In T-REx 293 cells (R78007, Thermo Fisher Scientific; mentioned as HEK293 in the text and figures) were cultured at 37°C in Dulbecco's modified Eagle's medium (DMEM) supplemented with 10% fetal bovine serum, 100 U/ml penicillin, and 100 μg/ml streptomycin. pcDNA5/FRT/TO_EGFP-L10a was co-transfected with pOG44 (V600520, Thermo Fisher Scientific) into Flp-In T-REx 293 cells to generate a stable cell line inducibly expressing EGFP-L10a and selected in media supplemented with 50 μg/ml hygromycin according to the manufacturer's instructions. EGFP-L10a expression was induced for 24 h by the addition of doxycycline at 3 μg/ml. Zebrafish were grown at 28°C in E3 embryo medium (5 mM NaCl, 0.17 mM KCl, 0.33 mM CaCl_2_ and 0.33 mM MgSO_4_) using standard methods (38). *Drosophila* S2 cells were maintained in Schneider's *Drosophila* medium (21720024, Thermo Fisher Scientific), supplemented with 10% fetal bovine serum and 1× antibiotic-antimycotic (15240096, Thermo Fisher Scientific) at 27°C. Preparation of synchronized KH1668 *smg-2* (*yb979*) worms were prepared as previously described (39). Briefly, synchronized gravid worms were cultivated in S-complete medium supplemented with *E. coli* strain OP50 and bleached with a standard bleach solution (40). The embryos were harvested and washed three times with M9 buffer. The embryos were then incubated in M9 buffer for 18 h at 20°C with gentle agitation for hatching, and L1 larvae were harvested and washed three times with M9 buffer.

### Immunoprecipitation of ribosome

HEK293 cells were seeded in a 6-well plate at 2.0 × 10^5^ cells per well. After 24 h, the cells were lysed in 200 μl of lysis buffer (20 mM HEPES-NaOH pH 7.5, 2.5 mM MgCl_2_, 150 mM NaCl, 1% (v/v) Triton X-100, 0.5 mM DTT, protease inhibitor cocktail, phosphatase inhibitor cocktail, 100 μg/ml cycloheximide). The cell lysates were clarified by centrifugation at 20,000 × *g* at 4°C for 10 min. The supernatant was mixed with 0.1 μg/μl RNase A or 1 unit/μl RNasin Plus and incubated with 1 μg of anti-ribosomal protein P0 antibody (isotype: mouse IgG2a κ, clone:9D5) (RN004M, MBL) or isotype control antibody (65208-1-IG, ProteinTech) at 4°C for 60 min. 7 μl of Dynabeads Protein G (10003D, Thermo Fisher Scientific) was added to the mixture and incubated at 4°C for 40 min. The beads were washed three times with 1 ml of wash buffer (20 mM HEPES-NaOH pH 7.5, 2.5 mM MgCl_2_, 150 mM NaCl, 0.05% (v/v) Tween 20) and directly mixed with 30 μl of SDS-PAGE sample buffer (20 mM HEPES-NaOH pH 7.5, 150 mM NaCl, 1% (v/v) SDS, 50 mM DTT), heated at 95°C for 5 min, and analyzed by western blotting. All proteins were detected using an ECL select western blotting detection reagent (RPN2235, cytiva) or ImmunoStar LD (296-69901, Wako) and imaged using an ImageQuant LAS 4000 mini (cytiva).

For immunoprecipitation of ribosomes from *Drosophila* S2 cells, 1.5 × 10^6^ cells were harvested by centrifugation at 600 × *g* for 5 min. The cell pellets were lysed in 400 μl lysis buffer (50 mM HEPES-NaOH pH 7.5, 10 mM MgCl_2_, 150 mM NaCl, 1% (v/v) NP-40, 0.5 mM DTT, protease inhibitor cocktail, phosphatase inhibitor cocktail, 100 μg/ml cycloheximide). The lysates were clarified by centrifugation at 20,000 × *g* for 10 min at 4°C. The supernatant was divided into two fresh microcentrifuge tubes and incubated with 1 μg of 9D5 antibody or isotype control IgG at 4°C for 60 min. The subsequent procedures were performed as described above.

For immunoprecipitation of the ribosome from zebrafish, 30 whole zebrafish embryos (4 days after fertilization) were resuspended in 1 ml lysis buffer (50 mM HEPES-NaOH pH 7.5, 100 mM KCl, 12 mM MgCl_2_, 1% (v/v) NP-40, 0.5 mM DTT, protease inhibitor cocktail, phosphatase inhibitor cocktail, 100 μg/ml cycloheximide, 1 mg/ml heparin, 0.2 unit/μl RNasin) and homogenized by douncing on ice. The lysates were then clarified by centrifugation at 10,000 × *g* at 4°C for 10 min. The supernatant was divided into two fresh microcentrifuge tubes and incubated with 1 μg of 9D5 antibody or isotype control IgG at 4°C for 60 min. Subsequent procedures were performed as described.

For immunoprecipitation of the ribosomes from the worms, synchronized L1 larvae were harvested by centrifugation at 600 x *g* for 1 min. The worm pellets were washed twice with 5 ml of M9 buffer and resuspended in 1 ml worm lysis buffer (50 mM HEPES-NaOH pH 7.5, 10 mM MgCl_2_, 150 mM NaCl, 1% (v/v) NP-40, 0.5 mM DTT, protease inhibitor cocktail, phosphatase inhibitor cocktail, 100 μg/ml cycloheximide). After homogenization by sonication, the lysates were clarified by centrifugation at 20,000 × *g* at 4°C for 10 min. The supernatant was divided into two fresh microcentrifuge tubes and incubated with 1 μg of 9D5 antibody or isotype control IgG at 4°C for 60 min. The subsequent procedures were performed as described above.

### ER stress and 4sU metabolic RNA labeling

The HEK293 cell line used for the inducible expression of EGFP-L10a was seeded in a 10 cm dish at 5.0 × 10^5^ cells per dish. The expression of EGFP-L10a was induced by 3 μg/ml of doxycycline for 24 h. The next day, the medium was quickly replaced with a freshly prepared medium containing 200 μM 4sU with either 0.02% (v/v) DMSO or 1 μM thapsigargin. The cells were incubated at 37°C for 3 h and lysed in 1 ml lysis buffer (20 mM HEPES-NaOH pH 7.5, 2.5 mM MgCl_2_, 150 mM NaCl, 1% (v/v) Triton X-100, 0.5 mM DTT, protease inhibitor cocktail, phosphatase inhibitor cocktail, 100 μg/ml cycloheximide). Whole cell lysates were clarified by centrifugation at 10,000 x *g* at 4°C for 10 min, and the supernatant supplemented with 1 unit/μl RNasin Plus was used for further procedure. For cytosolic RNA preparation, 200 μl of the supernatant was mixed with 500 μl of ISOGEN II (311-07361, NIPPON GENE) supplemented with 1 mM DTT and the prepared sample solution was stored at –20°C. For P-TRAP, 400 μl of the supernatant were incubated with 4 μg of 9D5 antibody at 4°C for 60 min. Afterward, 28 μl of Dynabeads Protein G was added to the mixture and incubated at 4°C for 40 min. After the beads were washed three times with wash buffer (20 mM HEPES-NaOH pH 7.5, 2.5 mM MgCl_2_, 150 mM NaCl, 0.05% (v/v) Tween 20), the beads were directly mixed with 500 μl of ISOGEN II supplemented with 1 mM DTT and the sample solution was stored at –20°C. For EGFP-L10a TRAP, 400 μl of the supernatant were incubated with 25 μl of GFP-Trap^®^ Magnetic Particles M-270 (gtd-100, proteintech) at 4°C for 60 min. After the beads were washed three times with wash buffer, the beads were directly mixed with 500 μl of ISOGEN II supplemented with 1 mM DTT, and the sample solution was stored at –20°C. The manufacturer's instructions were followed for RNA extraction using ISOGEN II, and the RNA samples were assessed for quality and quantity on MultiNA capillary electrophoresis instrument (Shimadzu).

### SLAMseq and QuantSeq

Cytosolic and immunoprecipitated RNAs were processed according to the standard SLAMseq protocol described previously (9). In brief, 5 μg of cytosolic RNA or immunoprecipitated RNA were incubated in 50 μl of alkylation buffer (50 mM NaPO_4_ buffer pH 8.0, 50% (v/v) DMSO, 10 mM iodoacetamide) at 50°C for 15 min. The reaction was quenched by adding 1 μl of 1 M DTT, followed by ethanol precipitation. For each sample, 500 ng of cytosolic RNA or immunoprecipitated RNA was used as an input for QuantSeq 3′ mRNA-Seq Library Prep Kit FWD for Illumina (Lexogen). The cDNA library was prepared according to the manufacturer's instructions. Libraries were assessed for quality using a MultiNA capillary electrophoresis instrument (Shimadzu), multiplexed to equimolar concentrations, and sequenced using the HiSeq 2500 system (Illumina) in PE-150 mode with a yield of 20–30 million reads per sample.

### Bioinformatic analysis

Total and nascent RNAs were quantified based on sequence data using SLAM-DUNK v 0.4.2, a pipeline for SLAMseq data analysis, with default parameters (9,41). Briefly, 12 bases from the 5′ end were trimmed as adaptor-clipped reads (-trim-5p 12), and then five or more subsequent adenines from the 3′ end were regarded as the remaining poly(A) tail and removed (-max-polya 4). Up to 100 regions with multiple mapped reads were allowed (-topn 100). As QuantSeq mainly targets the 3′ end of individual RNAs in a poly(A) tail-dependent manner, the sequence data were aligned on genome-wide 3′ UTR sequences generated based on the human genome sequence (GRCh38.p13) and annotation data (gencode.v41.annotation). Differential gene expression analysis was performed using DESeq2 v 1.3.8 (42). Differentially transcribed genes and differentially translated genes were identified using the deltaTE method (43). For DESeq2 and deltaTE analyses, we used the ReadCount (for total RNA) and TcReadCount (for nascent RNA) columns of the *tcount* file (output from SLAM-DUNK) as input. To analyze the TcReadCount column with the DESeq2 and deltaTE method, we calculated global normalization factors using the ReadCount column. The proportion of nascent transcripts to the total transcripts was estimated by normalizing the number of T>C reads to the total reads. Gene ontology analysis was performed by over-representation analysis using a biological process database (WebGestalt 2019) (44).

## RESULTS

### Combination of TRAP with 4sU metabolic RNA labeling for simultaneous measurement of nascent transcripts and their translation

**Figure 1.**
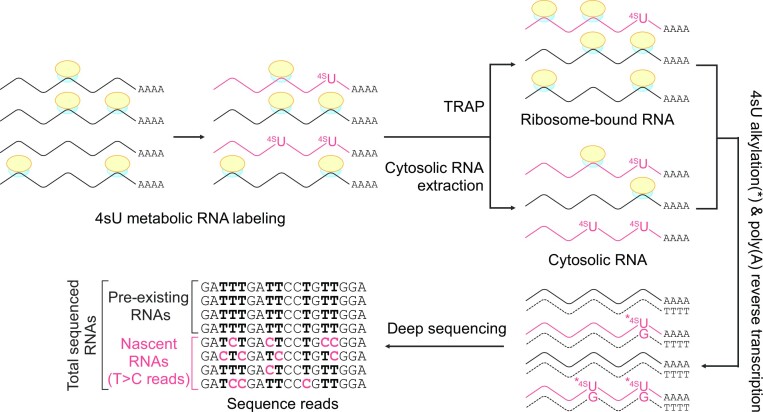
Schematic representation of the procedure for combing 4sU metabolic RNA labeling using the TRAP technique. Cultured cells are incubated with 4-thiouridine (4sU) for a defined period of time. Cells are lysed in a lysis buffer containing cycloheximide to prevent ribosome run-off and divided into cytosolic RNA and TRAP fractions. RNAs are reverse transcribed from poly(A) tail according to QuantSeq (3′ mRNA sequence) for cDNA library preparation and deep sequencing. Nascent RNAs are distinguished from total RNAs by the detection of T>C conversion at sites of 4sU incorporation.

SLAMseq distinguishes 4sU-labeled nascent transcripts from pre-existing RNA prior to labeling by bioinformatics detection of specific T>C conversions at the sites of 4sU incorporation. The combination of SLAMseq with QuantSeq allows the robust quantification of T>C conversions (9). As QuantSeq requires reverse transcription from poly(A), it is compatible with polysome profiling and TRAP techniques for recovering polyadenylated RNAs bound to ribosomes. Polysome profiling allows for the fractionation of monosomes and polysomes using a sucrose density gradient to monitor active translation. However, it requires specialized equipment (e.g. ultracentrifuges and gradient fractionation systems), is labor-intensive, and does not allow the handling of many samples in parallel (24). To ensure experimental throughput, we designed a combination of TRAP and SLAMseq/QuantSeq (Figure 1).

### Affinity purification of endogenous translating ribosome from human cultured cells using an antibody against ribosomal P-stalk

**Figure 2.**
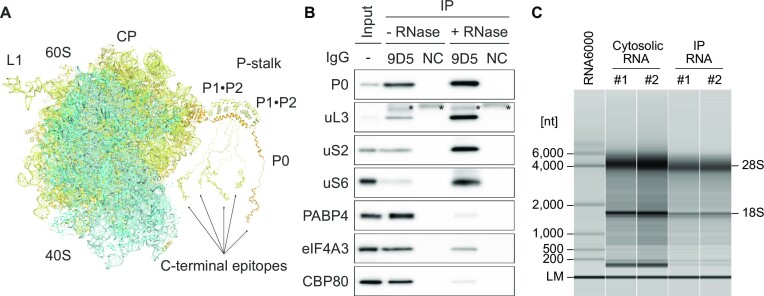
Immunoprecipitation of endogenous translating ribosomes mediated by the C-terminal IDRs of the P-stalk. (**A**) A structural model of human 80S ribosome containing ribosomal P-stalk generated from three coordinates [Protein data bank code: 3A1Y, 4BEH and 6EK0 (36,52,53)]. The C-terminal IDR of P0 is modeled arbitrarily. The epitopes of the anti-P0 antibody (9D5) are shown as a stick model. Colors are as follows: 40S ribosomal subunit (cyan), 60S ribosomal subunit (pale yellow), and ribosomal protein P0 (orange). (**B**) Immunoprecipitation of the endogenous ribosome and ribosome-bound RNA with the anti-ribosomal protein P0 antibody (9D5) or isotype control IgG (NC) from the cytosolic lysate of HEK293 cell in the presence or absence of RNase A, followed by western blotting. The input contained 1% of the lysate used for immunoprecipitation. Asterisks (*) indicate non-specific signals from the antibodies (9D5 or NC). (**C**) Examples of capillary electrophoresis profiles for cytosolic RNAs and immunoprecipitated RNAs from HEK293 cells. Two independent experiments were performed for each condition (#1, #2). The lower marker (LM) indicates internal standards (25 nt). RNA ladder (the Agilent RNA 6000 Pico kit) was used as a size marker.

TRAP requires genetically engineered cells expressing affinity-tagged ribosomal proteins of the large ribosomal subunit to purify translating ribosome-bound RNAs. The expression of the affinity-tagged ribosomal proteins with specific promoters enables the measurement of cell-, tissue- and developmental stage-specific translatome (22,27–29), which is a major advantage of TRAP. However, the genetic manipulation of endogenous ribosomal proteins reduces experimental throughput and is unsuitable for cells that cannot be genetically engineered. Therefore, we aimed to develop a high-throughput system that allows affinity purification of endogenous non-tagged ribosomes from various cells and organisms. To this end, we focused on the ribosomal P-stalk which is a multimeric ribosomal protein complex in the eukaryotic 60S ribosomal subunit. The P-stalk consists of one copy of ribosomal protein P0 and two heterodimers of ribosomal proteins P1 and P2, forming a pentametric P0-(P1-P2)_2_ complex (45). P0, P1 and P2 share homologous C-terminal intrinsically disordered regions (IDRs); therefore, the P-stalk contains five copies of C-terminal IDRs (46). As the homologous C-terminal IDRs of the P-stalk are exposed outside the ribosome, the antibody that recognizes the C-terminal IDRs of the P-stalk is expected to be suitable for the immunoprecipitation of endogenous ribosomes (Figure 2A) (47). Metz *et al.* recently applied an endogenous ribosome immunoprecipitation approach using 5.8S rRNA as an epitope as well (48). Besides, our method uses multiple epitopes in the ribosome instead of a single epitope for efficient immunoprecipitation.

We tested whether translating ribosomes could be immunoprecipitated from cultured human cell extracts using a monoclonal antibody (9D5) that recognizes the C-terminal IDRs of P0, P1 and P2 (49). The isotype control of 9D5 was used as a negative control for immunoprecipitation. As expected, the 9D5 antibody immunoprecipitated with ribosomal proteins uL3, uS2 and uS6, and mRNA-binding proteins CBP80, eIF4A3 and PABP4 (Figure 2B). RNase A treatment markedly decreased the intensity of CBP80, eIF4A3 and PABP4 but not that of ribosomal proteins, revealing that these mRNA-binding proteins were co-immunoprecipitated via ribosome-bound mRNA. In addition, the RNA electrophoresis profiles confirmed that representative ribosomal RNA signals (28S and 18S) and smear patterns of ribosome-bound RNAs were obtained by co-immunoprecipitation (Figure 2C). These results indicate that the 9D5 antibody against the ribosomal P-stalk enables the purification of endogenous non-tagged ribosomes and ribosome-bound RNAs.

### Affinity purification of endogenous ribosome via ribosomal P-stalk from diverse eukaryotes

**Figure 3.**
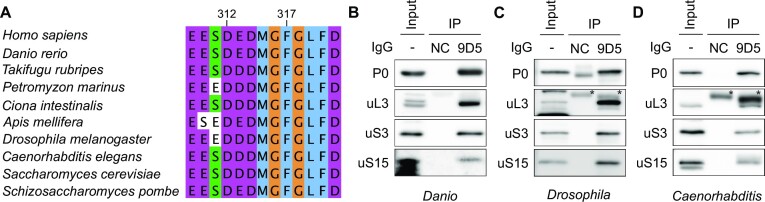
Immunoprecipitation of the endogenous ribosome from diverse eukaryotes mediated by the C-terminal IDRs of the P-stalk. (**A**) Sequence alignment of the C-terminal 12 amino acids of ribosomal protein P0 in diverse eukaryotes. The 9D5 anti-P0 antibody recognizes these sequences. (B–D) Immunoprecipitation of the endogenous ribosome with the 9D5 antibody or isotype control IgG (NC) from whole zebrafish embryo (**B**), Drosophila S2 cells (**C**) and adult C. elegans (**D**) in the presence of RNase A, followed by western blotting. The input contained 1% of the lysate used for immunoprecipitation. Asterisks (*) indicate non-specific signals from the antibodies (9D5 or NC). The amino acid numbers for human P0 are shown above.

A C-terminal 22 amino acids sequence in the IDRs of the P-stalk recognized by the 9D5 antibody is well-conserved in diverse eukaryotes from yeast to humans (Figure 3A). Therefore, the immunoprecipitation of endogenous ribosomes using the 9D5 antibody may be applied to diverse eukaryotes. We next tested whether the immunoprecipitation of endogenous ribosomes using the 9D5 antibody could be performed in zebrafish, fruit flies, and worms (Figures 3B–D). In these experiments, the 9D5 antibody immunoprecipitated with the ribosomal proteins uL3, uS3, and uS15, indicating that the 9D5 antibody can be used to purify endogenous 80S (translational) ribosomes from diverse eukaryotes without genetic manipulation. We termed the anti-P0 antibody-mediated purification of endogenous ribosomes as P-stalk-mediated translational ribosome affinity purification (P-TRAP).

### Simultaneous measurement of nascent transcriptome and translatome in the UPR by combining SLAMseq/QuantSeq with P-TRAP **or conventional L10a-**TRAP

We combined SLAMseq/QuantSeq with TRAP to analyze nascent RNAs and their translation at the level of individual genes in the unfolded protein response (UPR). In the UPR, transcription and translation are dynamically reprogrammed to reduce unfolded and misfolded proteins in the ER and restore protein homeostasis (50,51). To compare P-TRAP with conventional TRAP, we generated a HEK293 cell line for the stable expression of doxycycline-inducible enhanced green fluorescent protein (EGFP) fused with the N-terminus of ribosomal protein L10a (EGFP-L10a), an initially reported TRAP technique in mammals (Supplementary Figure S1A) (22,27–29). The expression of EGFP-L10a enabled the affinity purification of translating ribosomes containing EGFP-L10a using GFP nanobody-conjugated magnetic beads (Supplementary Figures S1B, C). We subjected the EGFP-L10a-expressing cells with 4sU for metabolic RNA labeling and thapsigargin, which inhibits the sarcoplasmic/endoplasmic reticulum calcium ATPase (SERCA) and leads to the accumulation of unfolded proteins in the ER for 3 h (52). The cell lysate was divided into three factions: cytosolic RNA (fraction I), ribosome-bound RNAs obtained by P-TRAP (fraction II), and conventional EGFP-L10a-TRAP (fraction III), followed by the QuantSeq (here we termed cytosolic RNA-seq, P-TRAP-seq and L10a-TRAP-seq). Principal component analysis (PCA) revealed that cytosolic RNA-seq, P-TRAP-seq, and L10a-TRAP-seq were distinguishable in the DMSO- and thapsigargin-treated groups (Supplementary Figure S2). In particular, the cytosolic RNA group was separated from the other two groups in PC1 (46.5% and 49.1% for total and T>C reads, respectively) and PC2 (15.8% and 8.3% for total and T>C reads, respectively), reflecting a low correlation between the transcriptome characterized by standard RNA-seq and the translatome (4,19–21) both in the presence or absence of thapsigargin. The similar trend was also confirmed by calculating correlation coefficients based on counts per million (CPM) for each method (Supplementary Figure S3).

### Translatome analysis in the UPR by P-TRAP-seq and L10a-TRAP-seq

**Figure 4.**
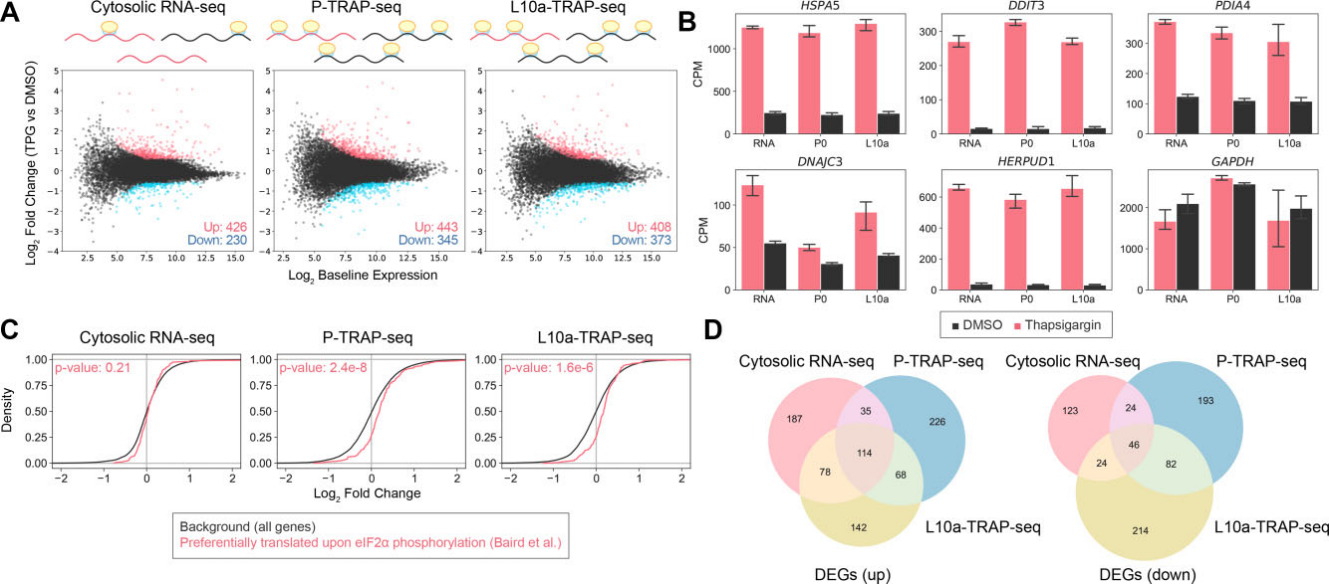
Transcriptome and translatome changes in the UPR at the total reads analysis. (**A**) MA plot of cytosolic RNA-seq, P-TRAP-seq and L10a-TRAP-seq data from thapsigargin (TPG)-treated versus DMSO-treated HEK293 cells. Differentially expressed genes (adjusted *P*-value < 0.05 and log_2_ fold change > |1.5|) are highlighted in red (up-regulated) or blue (down-regulated). (**B**) Counts per million (CPM) values of selected ER stress response genes (*HSPA5*, *DDIT3*, *PDIA4*, *DNAJC3* and *HERPUD1*) and a control gene (*GAPDH*) in the cytosolic RNA-seq (RNA), P-TRAP-seq (P0) and L10a-TRAP-seq (L10a) in response to DMSO- (black) or TPG- (red) treatments. The means and standard deviations of three replicates are shown. (**C**) Cumulative distributions of the log_2_ fold changes of selected gene categories (preferentially translated by polysome upon eIF2α phosphorylation) compared to all genes, together with *P*-values of Mann–Whitney *U* test. (**D**) Venn diagrams overlapping DEGs detected by the cytosolic RNA-seq (red), P-TRAP-seq (blue), and L10a-TRAP-seq (yellow) in the total reads analysis.

To validate whether translatome analysis with P-TRAP-seq and L10a-TRAP-seq captured gene expression reprogramming in the UPR, we performed differential gene expression analysis using total reads (Figure 4A, Supplementary Table S1) to identify differentially expressed genes (DEGs). In comparison to cytosolic RNA-seq, more DEGs were detected in P-TRAP-seq and L10a-TRAP-seq (656 genes in cytosolic RNA-seq, 788 genes in P-TRAP-seq, and 781 genes in L10a-TRAP-seq). The expression of typical genes involved in the UPR (*HSPA5*, *DDIT3*, *PDIA4*, *DNAJC2* and *HERPUD1*) was significantly increased in P-TRAP-seq analysis similar to cytosolic RNA-seq and conventional L10a-TRAP-seq (Figure 4B). Notably, specific genes preferentially translated in the UPR as determined by polysome profiling (53) were up-regulated in the P-TRAP-seq and L10a-TRAP-seq but not in the cytosolic RNA-seq (Figure 4C). These results indicate that P-TRAP-seq detects changes in translation without changing the RNA abundance and is well suited for translatome analysis like the conventional L10a-TRAP method and polysome profiling.

Although not as significant as the difference between transcriptomes and translatomes, P-TRAP-seq and L10a-TRAP-seq were separated in the PCA analysis (Supplementary Figure S2). This suggested that P-TRAP-seq and L10a-TRAP-seq may have captured slightly different RNA subsets. Indeed, the DEGs identified by P-TRAP-seq and L10a-TRAP-seq showed incomplete overlap, with numerous genes uniquely detected by one of the two protocols (Figure 4D). This discrepancy likely reflects ribosomal heterogeneity and consequent differences in the RNA subsets captured by each technique (see Discussion).

### Categorical analysis of nascent transcriptome and translatome in the UPR

**Figure 5.**
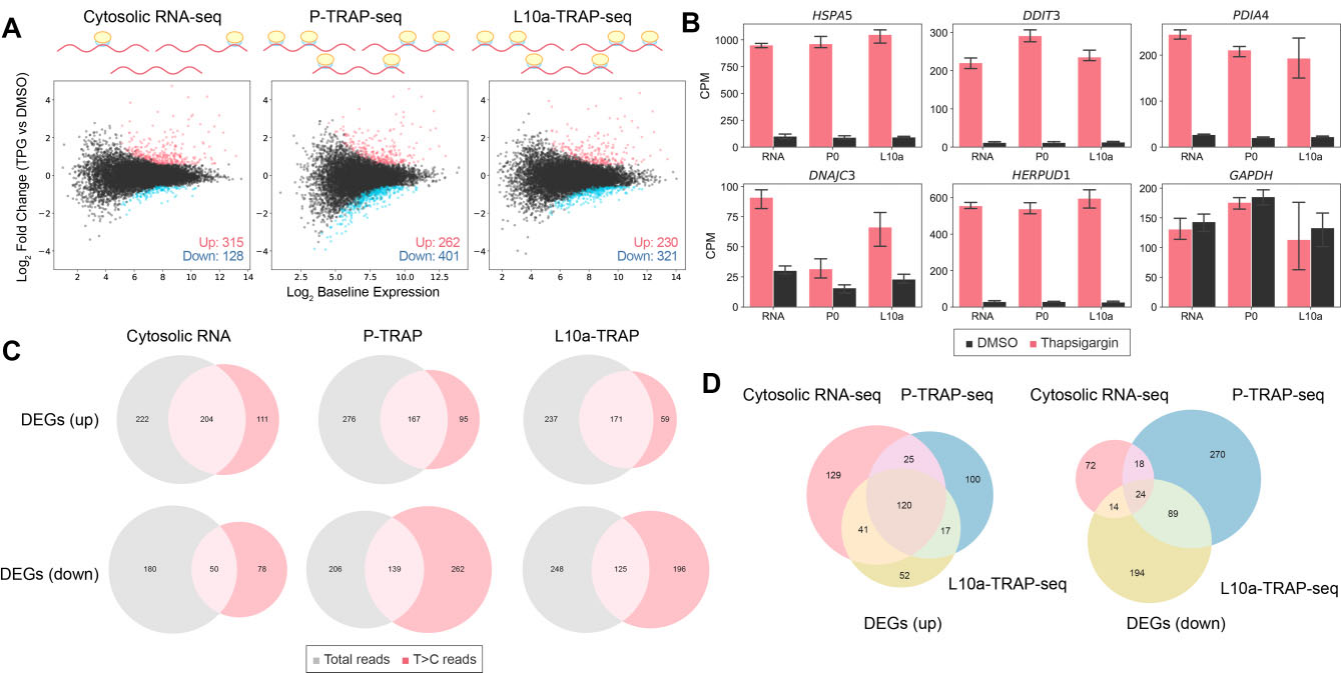
Transcriptome and translatome changes in the UPR at the T>C reads analysis. (**A**) MA plot of cytosolic RNA-seq, P-TRAP-seq and L10a-TRAP-seq data from thapsigargin (TPG)-treated versus DMSO-treated HEK293 cells. Differentially expressed genes (adjusted *P*-value < 0.05 and log_2_ fold change > |1.5|) are highlighted in red (up-regulated) or blue (down-regulated). (**B**) Counts per million (CPM) values of selected ER stress response genes (*HSPA5*, *DDIT3*, *PDIA4*, *DNAJC3* and *HERPUD1*) and a control gene (*GAPDH*) in the cytosolic RNA-seq (RNA), P-TRAP-seq (P0), and L10a-TRAP-seq (L10a) in response to DMSO- (black) or TPG- (red) treatments. The means and standard deviations of three replicates are shown. (**C**) Venn diagrams overlapping DEGs detected by the cytosolic RNA-seq, P-TRAP-seq and L10a-TRAP-seq between the total reads analysis (grey) and the T>C reads analysis (red). (**D**) Venn diagrams overlapping DEGs detected by the cytosolic RNA-seq (red), P-TRAP-seq (blue), and L10a-TRAP-seq (yellow) in the T>C reads analysis.

In SLAMseq, 4sU incorporated into nascent RNAs is alkylated during library preparation, and T>C conversion occurs via reverse transcription. Consistent with a previous report (9), we observed a median rate of 2.3–2.7% for T>C conversion and < 0.2% for any other conversion in total RNA in the presence of 200 μM 4sU (Supplementary Figure S4). We extracted sequence reads with T>C conversion (T>C reads) using the SLAM-DUNK pipeline and obtained the nascent transcriptome (cytosolic RNA-seq) and nascent translatome (P-TRAP-seq and L10a-TRAP-seq) during the UPR. Differential gene expression analysis of nascent RNAs revealed 443, 663 and 551 DEGs in the cytosolic RNA-seq, P-TRAP-seq and L10a-TRAP-seq, respectively (Figure 5A, Supplementary Table S1). Similar to the total reads analysis, the expression of typical genes involved in the UPR increased in the nascent transcriptome and translatome (Figure 5B). Up-regulated DEGs detected by RNA-seq, P-TRAP-seq, and L10a-TRAP-seq in the nascent RNA analyses were less than those in the total RNA analysis. On the other hand, down-regulated DEGs were detected to be in less quantity by RNA-seq as compared to detection by P-TRAP-seq and L10a-TRAP-seq (Figure 5C). In the total reads analysis (Figure 4D), the DEGs identified by P-TRAP-seq and L10a-TRAP-seq showed incomplete overlap, with numerous genes uniquely detected by one of the two methods (Figure 5D).

The different ratios of up- and down-regulated DEGs in the total reads analysis to the T>C reads analysis in the transcriptome and translatome suggest that the regulation of nascent RNAs and pre-existing RNAs is different between the transcriptome and translatome (Figure 5C). To investigate the regulation of nascent RNAs and pre-existing RNAs in the transcriptome and translatome, we anticipated that it would be effective to subtract T>C reads from total reads to obtain non-T>C reads which may reflect the abundance of pre-existing RNA. Importantly, the non-T>C reads are expected to include some reads that are derived from nascent RNAs but do not contain T>C conversion. If the proportion of non-T>C reads derived from these nascent RNAs is large, then the estimation of the proportion of pre-existing RNAs based on the subtracted read counts will be inaccurate. To evaluate the sensitivity of the detecting of T>C reads under the present experimental conditions, we focused on the three genes (*CHAC1*, *DDIT3* and *HERPUD1*) that showed the highest ratio of increased RNA levels after the addition of 4sU and thapsigargin in the cytosolic RNA-seq. In these genes, most reads increased after the addition of 4sU and thapsigargin could be considered to be derived from nascent RNA. By calculating the proportion of reads detected as T>C reads out of all these reads, we estimated the sensitivity of detecting T>C reads under the present experimental conditions and determined that approximately 82–86% of the reads derived from nascent RNAs were detected as T>C reads (Supplementary Figures S5A and B).

**Figure 6.**
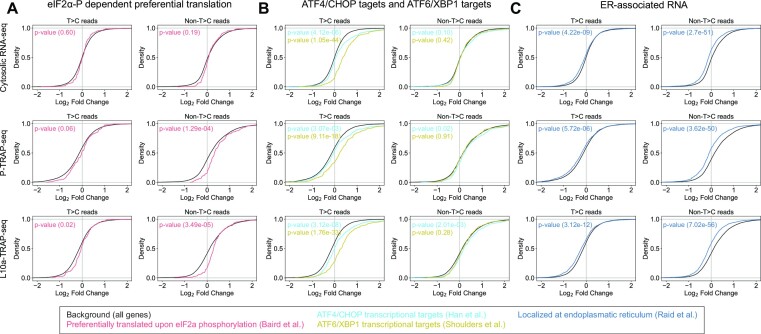
Categorical gene expression reprogramming of the T>C reads and non-T>C reads analyses. Cumulative distributions of the log_2_ fold changes of selected gene categories, preferentially translated by polysome upon eIF2α phosphorylation (**A**), ATF6/XBP1 transcriptional targets (**B**) and localized at the ER, ATF4/CHOP transcriptional targets (**C**) compared to all genes, together with *P*-values of Mann–Whitney *U* test.

In the acute phase of the UPR, the ER stress sensor PERK phosphorylates translation initiation factor 2α (eIF2α) at serine 51, leading to global repression of translation initiation, and the whole protein synthesis rate is transiently reduced to 15–20% (54,55). The phosphorylation of eIF2α also promotes the preferential translation of mRNAs with upstream open reading frames (uORFs), such as transcription factors ATF4 and CHOP. In the non-T>C reads analysis, the RNAs enriched in the polysome fraction upon eIF2α phosphorylation were significantly up-regulated in both P-TRAP-seq and L10a-TRAP-seq, but not that in cytosolic RNA-seq (Figure 6A). In contrast, the elevated levels of these RNAs were relatively mild in the T>C reads analysis suggesting that the phosphorylated eIF2α-dependent preferential translation primarily occurs to pre-existing RNAs in the UPR.

We next analyzed gene expression reprogramming for the essential branches of the UPR, the ATF4 and CHOP pathway (ATF4/CHOP target) and the ATF6 and XBP1 pathway (ATF6/XBP1 target). Consistent with previous reports (56,57), the RNA levels of ATF4/CHOP and ATF6/XBP1 targets were significantly elevated in the T>C reads analysis upon thapsigargin treatment, but not in the non-T>C reads analysis (Figure 6B). These results are consistent with the enhanced transcriptional activity of ATF4/CHOP and ATF6/XBP1 during the UPR, and support that the non-T>C analysis could separate pre-existing RNAs from nascent RNAs in this experimental condition. The abundance of ATF6/XBP1 targets was also significantly elevated in nascent translatomes obtained by P-TRAP-seq and L10a-TRAP-seq, indicating that the nascent RNAs transcribed by ATF4/CHOP and ATF6/XBP1 were translated during the UPR (Figure 6B).

In the acute phase of the UPR, the total amount of proteins synthesized on the ER membrane is reduced to decrease protein loading to the ER (50,51). The expression of genes encoding membrane and secretory proteins was significantly downregulated in both the T>C reads analysis and the non-T>C reads analysis in the cytosolic RNA-seq data in response to ER stress (Figure 6C). Notably, the reduction in RNA levels localized to the ER membrane was more prominent in the non-T>C reads analysis than in the T>C reads analysis. Similar patterns were observed in the P-TRAP-seq and L10a-TRAP-seq data. These results indicate that the UPR likely destabilizes pre-existing RNAs localized to the ER membrane, known as the output of regulated IRE1-dependent decay (RIDD) (58,59).

### Analysis of nascent transcriptome and translatome in the UPR for individual genes

**Figure 7.**
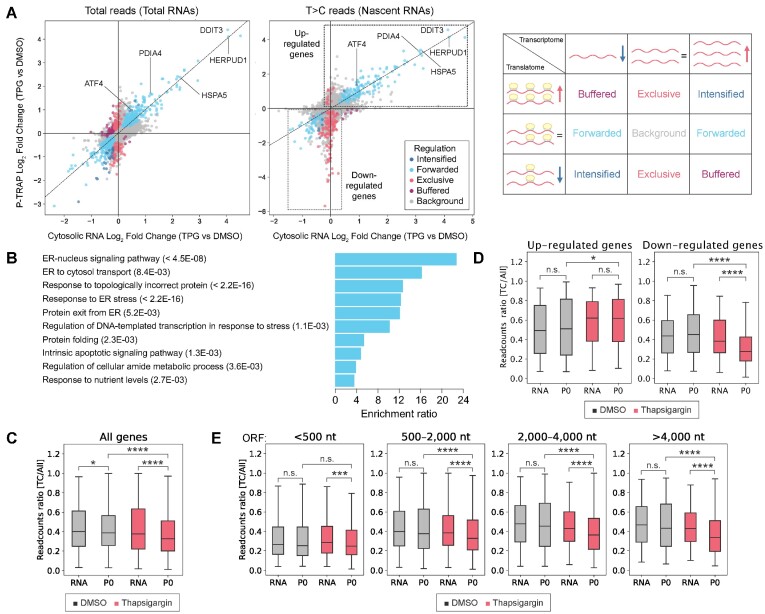
Translation regulation of nascent RNAs in the UPR. (**A**) Left: fold changes each total or nascent RNAs in cytosolic RNA-seq and P-TRAP-seq data at the levels of the individual genes analyzed by the deltaTE method (FDR < 0.05 and log_2_ fold change > |0|). Translationally forwarded genes (cyan), intensified genes (blue), exclusive genes (red) and buffered genes (purple) are highlighted. Right: Regulation modes categorized by deltaTE analysis (43). (**B**) The enrichment ratios in over-representation analysis (ORA) of up-regulated translationally forwarded genes in deltaTE analysis (T>C reads) performed by WebGestalt 2019 analysis (44). Resulting biological processes and FDR values are shown. (**C**) Box plots of the proportion of nascent RNAs at the level of bulk RNAs in the cytosolic RNA-seq (RNA) and P-TRAP-seq (P0) in response to DMSO- (black) or TPG- (red) treatment. n.s., not significant, *P < 0.05, ****P < 0.0001 (Mann–Whitney *U* test). (D, E) Box plots of the proportion of nascent RNAs in the groups of RNAs according to expression states (up-regulated genes and down-regulated genes) categorized by deltaTE analysis (**D**) or groups of RNAs according to ORF length (**E**). n.s., not significant, *P < 0.05, ***P < 0.001, ****P < 0.0001 (Mann–Whitney *U* test).

One of the advantages of combining TRAP-seq and SLAMseq/QuantSeq is the robust estimation of translation levels of nascent RNAs for individual genes as compared to nRibo-seq. In order to further investigate the translational states in the UPR, we performed deltaTE analysis (43) for total or nascent RNAs from cytosolic RNA-seq and P-TRAP-seq data (Figure 7A, Supplementary Table S2). The deltaTE algorithm classified genes into four categories: (i) genes regulated at the RNA level (forwarded), (ii) genes regulated at the translation level (exclusive), (iii) genes regulated by the synergistic effect of RNA and translation levels (intensified), and (iv) genes regulated by the levels of RNA and translation against each other (buffered). Consequently, deltaTE analysis revealed that the actively transcribed RNAs during UPR (categorized as forwarded) were loaded to the ribosomes indicating that these actively transcribed RNAs relatively escaped from the global repression of translation initiation by eIF2α phosphorylation (Figure 7B, expressed genes). These actively translated RNAs included genes involved in the UPR (*HSPA5*, *HERPUD1*, and *PDIA4*), and enrichment analysis showed significant enrichment of genes responsible for ER stress tolerance (Figure 7B). Notably, deltaTE analysis revealed that a certain subset of nascent RNAs still undergo translational repression (Figure 7A). These results are consistent with previous observations that the UPR leads to massive translational repression associated with eIF2α phosphorylation, followed by the synthesis of proteins involved in the proper folding or degradation of unfolded and misfolded proteins in the ER (60,61).

Previous study showed that there is a lag in the accumulation of nascent RNAs between the transcriptome and the translatome at steady-state condition (35). Therefore, changes in the nascent translatome in the UPR may be influenced by how rapidly nascent RNAs are loaded onto ribosomes. We estimated the proportion of nascent RNAs in the transcriptome and translatome by normalizing the T>C reads to total reads and investigated whether the UPR influences ribosomal loading of nascent RNAs. At the level of bulk RNAs, only slight differences were observed in steady-state condition (Figure 7C). However, a significant decrease in the proportion of nascent RNAs was observed in the translatome, in contrast to the transcriptome, indicating a reduced ribosomal loading of nascent RNAs in the UPR (Figure 7C). Notably, the proportion of nascent RNAs of the up-regulated genes during UPR was not significantly decreased in the translatome (Figure 7D, up-regulated genes). This indicated that ribosomes are rapidly loaded on these genes after transcription. On the other hand, the proportion of nascent RNAs of down-regulated genes was significantly reduced in the translatome, suggesting that ribosomal loading of these genes is decreased in the UPR (Figure 7D, down-regulated genes). These data suggest the existence of uncharacterized mechanisms, to escape or to be translationally repressed in certain gene subsets.

ORF length has been discussed as one of the parameters that determine the ribosomal loading rate (35,62). We investigated whether ORF length might influence the ribosomal loading of nascent RNAs. Although, no significant differences were detected in steady-state condition, thapsigargin treatment decreased the proportion of nascent RNAs in the translatome in an ORF length-dependent manner (Figure 7E). These results indicate that ORF length is one of the parameters that influences ribosomal loading of nascent RNAs in the UPR. Further analysis is required to understand the complexity of gene expression reprogramming in the UPR.

## DISCUSSION

In this study, we established a simple protocol for the simultaneous measurement of nascent RNAs and their translation at the gene level. Recently, Schott *et al.* developed nRibo-seq for the simultaneous measurement of nascent RNA synthesis and translation efficiency at the level of bulk RNAs or specific gene categories by combining Ribo-seq with 4sU metabolic RNA labeling (35). Instead of Ribo-seq, we combined TRAP-seq with 4sU metabolic RNA labeling. Because TRAP-seq is compatible with QuantSeq, enabling the accurate quantification of T>C conversions from a small number of sequence reads, it is suitable for the simultaneous measurement of nascent RNA synthesis and translation efficiency at the levels of individual genes. In addition, TRAP-seq does not require specialized equipment such as ultracentrifugation and gradient fractionation systems, is not labor-intensive, and enables the recovery of ribosome-bound RNAs from a small number of cells. These advantages of TRAP-seq make it compatible with automated library preparation systems for high-throughput sequencing procedures, such as single-cell RNA sequencing. Furthermore, in contrast to conventional TRAP-seq, which requires genetic manipulation for the affinity purification of ribosomes, the newly developed P-TRAP-seq method can be applied to immunoprecipitate endogenous ribosomes without genetic manipulation. Therefore, measuring nascent RNAs and their translation using P-TRAP-seq with 4sU metabolic RNA labeling, nascent-P-TRAP-seq (nP-TRAP-seq), is a simple and robust method for investigating the crosstalk between transcriptional and translational regulation. Furthermore, non-T>C reads may be analyzed as pre-existing RNAs under experimental conditions that ensure high detection sensitivity for T>C reads. As shown in the present study, we observed preferential translational reprogramming of both nascent and pre-existing RNAs in response to the UPR. Although individual translation efficiency can be monitored using mass-spectrometry-based techniques such as PUNCH-P (63) and BONCAT (64), our sequencing-based method is simple, low-cost, and provides highly accurate measurements.

It should be noted that the translatome measured using TRAP-seq only reflects ribosome-bound RNA levels. In contrast to Ribo-seq, TRAP-seq cannot determine the position of individual ribosomes in RNAs and has less resolution for complex translational regulation (e.g. translation efficiency for multiple open reading frames in a single transcript). TRAP-seq relies on a simple immunoprecipitation technique to extract ribosomes, including monosomes and polysomes; therefore, it cannot selectively fractionate actively translating polysomes, similar to the polysome profiling technique (65). Since the polysome profiling also yields full-length ribosome-bound RNA and is compatible with QuantSeq, a combination of polysome profiling and 4sU metabolic RNA labeling is preferred when analyzing more efficiently translated RNAs (i.e. RNAs bound to multiple ribosomes).

There are two major advantages to the immunoprecipitation of endogenous ribosomes with C-terminal IDRs of the ribosomal P-stalk. The first is the high specificity and efficiency of immunoprecipitation. The C-terminal IDRs of the P-stalk were present in five copies on the 60S large ribosomal subunit and exposed on the solvent side (Figure 2A). Although these IDRs bind to multiple translational GTPase factors (trGTPases), such as eEF1A and eEF2 (66), the 9D5 antibody binds preferentially and stably to the P-stalk rather than to trGTPases (49,67). In addition, because P-stalk incorporation into the pre-60S ribosomal subunit occurs in the late cytoplasmic stage of ribosome assembly, P-TRAP only captures mature translating ribosome particles (68,69). Therefore, antibodies recognizing the C-terminal IDRs of the P-stalk ensure stable, specific, and robust immunoprecipitation of endogenous translating ribosomes.

Secondly, the C-terminal IDRs of the P-stalk are conserved across most, if not all, eukaryotes (47). Indeed, antibodies that recognize the highly conserved C-terminal IDR allow the purification of endogenous ribosomes from cultured human cells, zebrafish, flies, and nematodes without any genetic manipulations (Figure 3). P-TRAP can shorten the experimental period compared with conventional TRAP, which requires genetic manipulation. Furthermore, immunoprecipitation of endogenous ribosomes in P-TRAP allows translatome analysis from non-model organisms without the need for genetic manipulation techniques or from human specimens in clinical research. Moreover, the immunoprecipitation of ribosomes using P-stalks can be applied to other experimental systems that require ribosome collection, such as Ribo-seq. Highly efficient ribosome collection without ultracentrifugation is expected to significantly reduce the time and effort required for experiments and simplify protocols. These advantages of P-TRAP will accelerate translatome research and contribute to our understanding of gene expression regulation in various biological fields such as UPR (50,51), nonsense-mediated mRNA decay (70), immune responses (71), and drug responses (44).

Both P-TRAP-seq and L10a-TRAP-seq captured the changes of the translatome in the UPR, but they identified several different DEGs (Figure 4D and 5C). Recent proteomic analyses have shown diversity in the composition of ribosomal proteins, referred to as ribosomal heterogeneity, resulting in the preferential translation of specific RNA. For example, L10a is present at substoichiometric levels in mouse embryonic stem cells (mESCs), and ribosomes containing L10a have an altered affinity for some transcripts (72). Performing TRAP-seq using substoichiometric ribosomal proteins as bait for immunoprecipitation may cause translatome bias. The P-stalk is present in polysomes with 1:1 stoichiometry in mESCs (72), indicating that the translatome obtained with P-TRAP-seq would have less translation bias due to ribosome heterogeneity, making the P-stalk an ideal bait for the TRAP-seq technique. However, in several experimental designs, P-TRAP has a disadvantage compared to the conventional TRAP technique because of the purification of ribosomes without genetic manipulation. Conventional TRAP captures gene expression from particular cells/tissues of genetically modified organisms expressing affinity-tagged ribosomal proteins using cell/tissue-specific promoters, such as the Gal4-UAS system in the fly or the Cre-lox system in mice (27,28,73). In contrast to conventional TRAP, P-TRAP cannot fractionate ribosomes from specific cells or tissues. Therefore, it is essential to use conventional TRAP and/or P-TRAP according to the experimental design.

## Supplementary Material

gkad545_Supplemental_Files

## Data Availability

The deep sequencing data reported in this study have been deposited in the DDBJ (DRA015587). All codes required to perform the computational analysis are available on Zenodo (https://zenodo.org/record/7943421).
