# Supplementary material for: Simultaneous measurement of nascent transcriptome and translatome using 4-thiouridine metabolic RNA labeling and translating ribosome affinity purification
Source: Nucleic Acids Res. 2023 Jun 28;51(14):e76. doi: 10.1093/nar/gkad545 (PMC10415123; doi:10.1093/nar/gkad545)
Supplement: gkad545_Supplemental_Files [file gkad545_supplemental_files.zip › Supplemental_Information_revised.pdf]

Simultaneous measurement of nascent transcriptome and translatome using 4-thiouridine metabolic RNA labeling and translating ribosome affinity purification

Hirotatsu Imai[1]*, Daisuke Utsumi[2], Hidetsugu Torihara[3], Kenzo Takahashi[2], Hidehito Kuroyanagi[3], Akio Yamashita[1]*

1. Department of Investigative Medicine, University of the Ryukyus, Okinawa, Japan
2. Department of Dermatology, University of the Ryukyus, Okinawa, Japan
3. Department of Biochemistry, University of the Ryukyus, Okinawa, Japan

*Corresponding authors:

Hirotatsu Imai (himai@med.u-ryukyu.ac.jp)

Akio Yamashita (akyama21@med.u-ryukyu.ac.jp)

1. Supplementary Figures

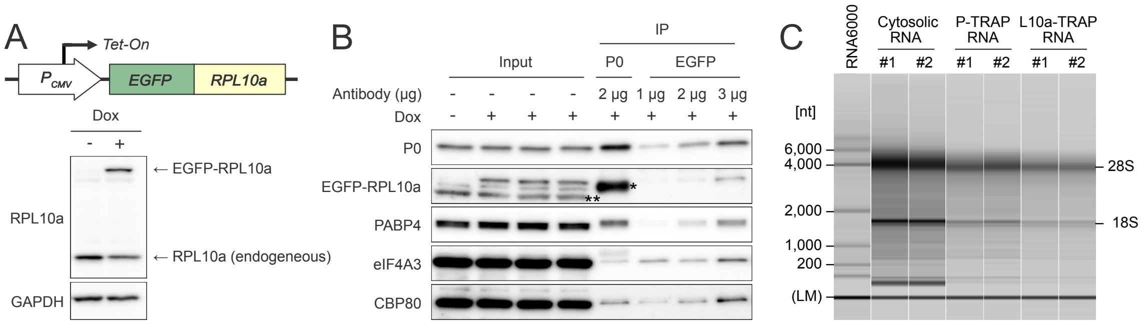

Supplementary Figure S1. (A) Top: schematic representation of HEK293 cell line stably expressing doxycycline-inducible (EGFP-RPL10a). Bottom: the doxycycline-inducible protein expression level of EGFP-RPL10a analyzed by western blotting. EGFP-RPL10a were detected by anti RPL10a antibody. (B) Immunoprecipitation of the endogenous ribosome and RNA binding proteins with the anti-ribosomal protein P0 antibody (9D5) or GFP-Trap® Magnetic Particles from the cytosolic lysate of HEK293 cell, followed by western blotting. The input contained 1% of the lysate used for immunoprecipitation. Asterisk (*) indicates a non-specific signal from antibodies (9D5). (C) Examples of capillary electrophoresis profiles for cytosolic RNA, P-TRAP RNA, and L10a-TRAP RNA from HEK293 cells. Two independent experiments were performed for each condition (#1, #2). The lower marker (LM) indicates internal standards (25 nt). RNA ladder (the Agilent RNA 6000 Pico kit) was used as a size marker.

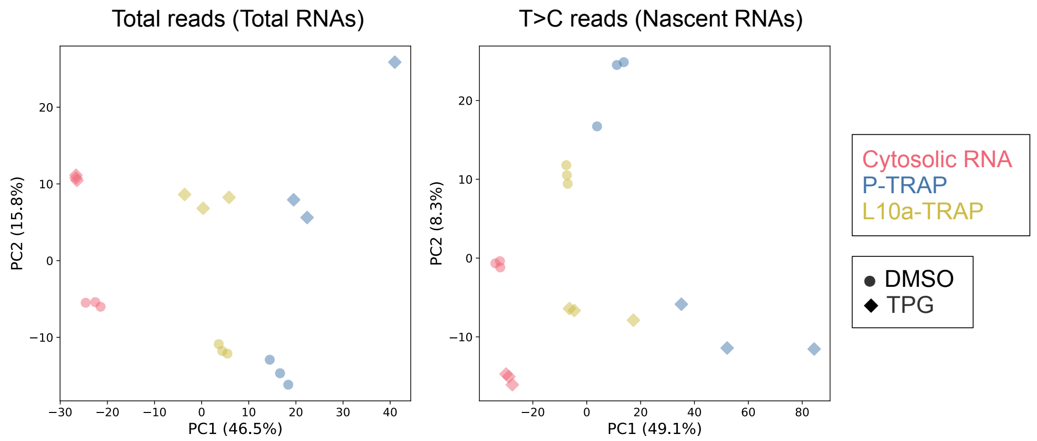

Supplementary Figure S2. Principle component analysis (PCA) of cytosolic RNA-seq (red), P-TRAP-seq (blue), and L10a-TRAP-seq (yellow) in the DMSO- (circle) and TPG-treated groups (diamond).

Figure S3

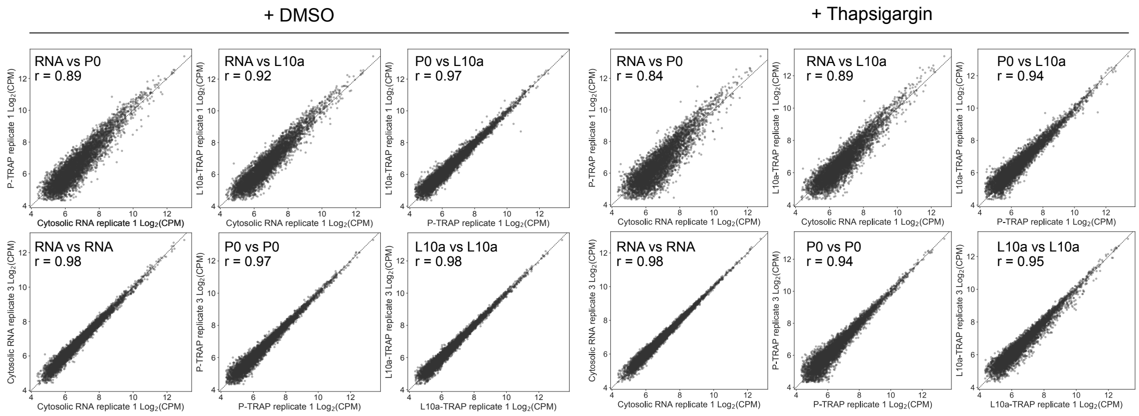

Supplementary Figure S3. Scatter plots comparing cytosolic RNA-seq, P-TRAP-seq and L10a-TRAP-seq. (A) For each gene, the log2 read counts per million (CPM) of cytosolic RNA-seq (RNA), P-TRAP-seq (P0), and L10a-TRAP-seq (L10a) were plotted against other or identical methods. We used replicate 1 for comparisons between different methods (top) and replicates 1 and 2 for comparisons between the same methods (bottom), respectively. Pearson correlation coefficients (r) are shown.

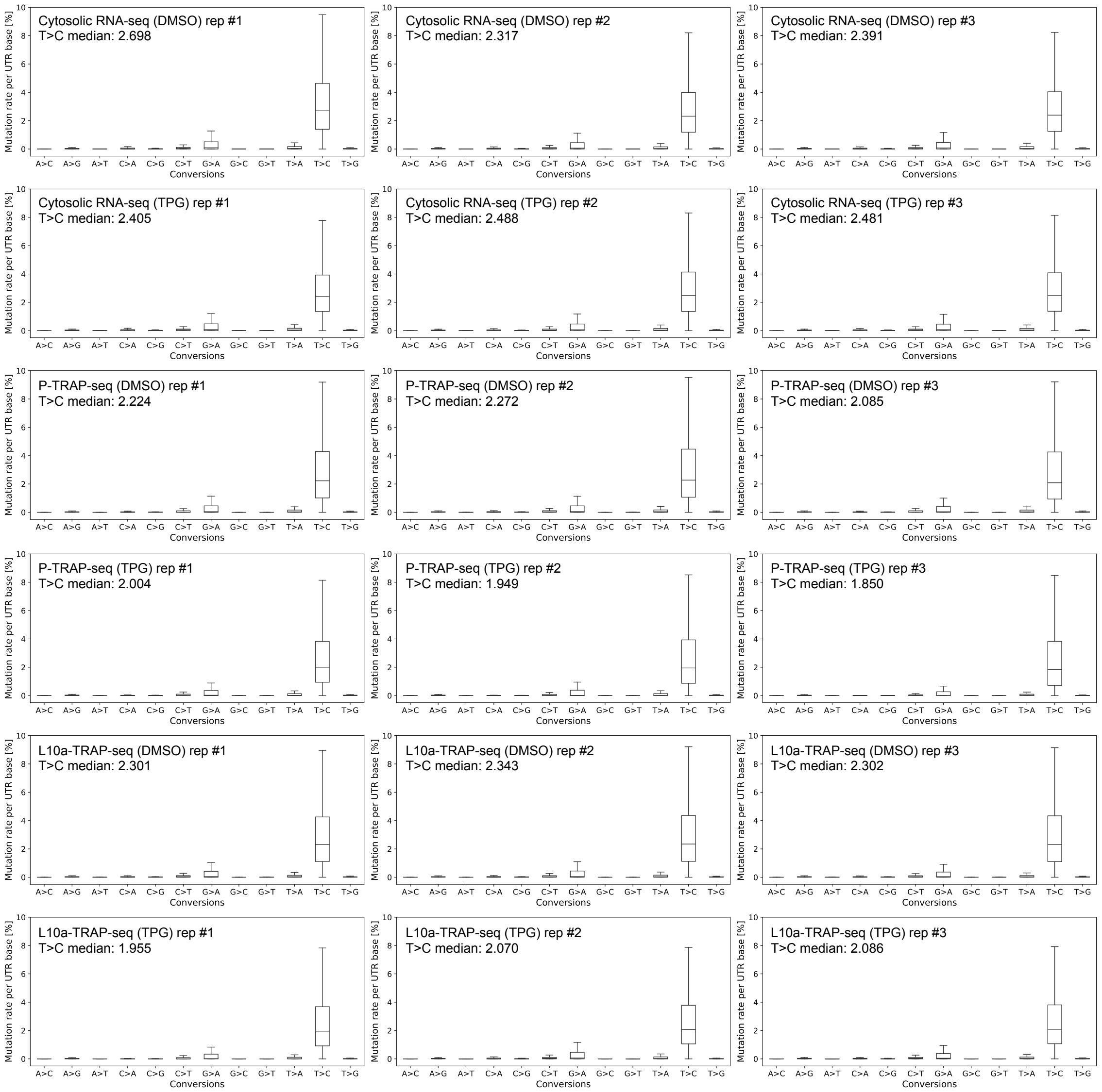

Supplementary Figure S4. Conversion rates in defined count window mapping reads of all QuantSeq libraries prepared from HEK293 cells after 3 hours of metabolic RNA labeling with 200 μM s4U.

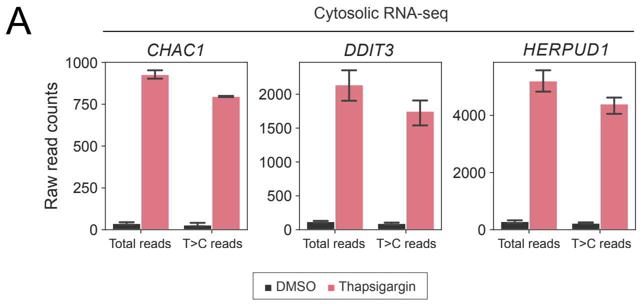

B

$$\text{T>C reads Proportion (in the UPR)} = \frac{\text{Mean(raw T>C read counts (TPG) − raw T>C read counts (DMSO))}}{\text{Mean(raw total read counts (TPG) − raw total read counts (DMSO))}}$$

Gene	T>C reads Proportion
CHAC1	0.82
DDIT3	0.86
HERPUD1	0.85

Supplementary Figure S5. (A) Raw total read counts and raw T>C read counts of selected ER stress response genes (*CHAC1*, *DDIT3*, and *HERPUD1*) in cytosolic RNA-seq in response to DMSO (black) or thapsigargin (TPG) (red) treatment. Means and standard deviations of three replicates are shown. (B) Calculation of the proportion of T>C reads out of the total reads increased by TPG treatment for these genes.

2. Supplementary Tables

Supplementary Table S1. Results of deferential gene expression analysis by DESeq2 for RNA-seq, P-TRAP-seq, and L10a-TRAP-seq.

Supplementary Table S2. Results of differentially transcribed genes and differentially translated genes analysis using deltaTE method for RNA-seq, P-TRAP-seq, and L10a-TRAP-seq.
